# Adverse childhood experiences and poor birth outcomes in a diverse, low-income sample

**DOI:** 10.1186/s12884-019-2560-8

**Published:** 2019-10-28

**Authors:** Joshua P. Mersky, ChienTi Plummer Lee

**Affiliations:** Institute for Child and Family Well-being, Helen Bader School of Social Welfare, University of Wisconsin-Milwaukee, 2400 E. Hartford Ave, Milwaukee, WI 53211 USA

**Keywords:** Adverse childhood experiences, Birth outcomes, Reproductive health, Pregnancy loss, Preterm birth, Low Birthweight, Low-income

## Abstract

**Background:**

Adverse childhood experiences (ACE) are associated with an array of health consequences in later life, but few studies have examined the effects of ACEs on women’s birth outcomes.

**Methods:**

We analyzed data gathered from a sample of 1848 low-income women who received services from home visiting programs in Wisconsin. Archival program records from a public health database were used to create three birth outcomes reflecting each participant’s reproductive health history: any pregnancy loss; any preterm birth; any low birthweight. Multivariate logistic regressions were performed to test the linear and non-linear effects of ACEs on birth outcomes, controlling for age, race/ethnicity, and education.

**Results:**

Descriptive analyses showed that 84.4% of women had at least one ACE, and that 68.2% reported multiple ACEs. Multivariate logistic regression analyses showed that cumulative ACE scores were associated with an increased likelihood of pregnancy loss (OR = 1.12; 95% CI = 1.08–1.17), preterm birth (OR = 1.07; 95% CI = 1.01–1.12), and low birthweight (OR = 1.08; 95% CI = 1.03–1.15). Additional analyses revealed that the ACE-birthweight association deviated from a linear, dose-response pattern.

**Conclusions:**

Findings confirmed that high levels of childhood adversity are associated with poor birth outcomes. Alongside additive risk models, future ACE research should test interactive risk models and causal mechanisms through which childhood adversity compromises reproductive health.

## Background

Adverse childhood experiences (ACEs) are a prevalent class of acute or recurring stressors that have long-lasting health consequences. Research in the U.S. has shown that most adults report at least one ACE, and that a higher number of ACEs increases the risk of various disorders and diseases in adulthood [1–6]. Due to their prevalence and influence, ACEs have come to be recognized as a major public health problem that should be monitored through population surveys such as the Behavioral Risk Factor Surveillance System [7].

ACEs like emotional neglect, physical abuse, and sexual abuse have been linked to an increased risk of poor birth outcomes such as pregnancy loss and preterm birth [8, 9]. Yet, despite the surge of ACE research over the last two decades, surprisingly few studies have examined the cumulative impact of ACEs on birth outcomes. Seminal findings from the Adverse Childhood Experiences Study did show that higher ACE scores were associated with an increased risk of fetal death [10]. Extending these findings, the National Child Development Study in Great Britain documented a positive graded relationship between a greater number of childhood hardships and the likelihood of preterm birth [11]. These results were reinforced by a recent Canadian study that found two or more ACEs roughly doubled the risk of preterm birth [12]. Although ACEs have been linked to preterm birth, and preterm birth is known to be a leading cause of low birthweight in developed countries [13], research on the ACE-birthweight connection has produced mixed results. Some studies have reported that greater childhood adversity increases the risk of low birthweight [11, 14], though one study found that birthweight was unrelated to the frequency of traumatic events [15].

Another lingering question in the literature pertains to whether a higher number of ACEs incrementally increases the probability of negative consequences. Research has frequently uncovered a linear, or dose-response, association between the number of ACEs and the risk of poor health outcomes. However, some studies have found that the effects of maltreatment and other adversities follows a non-linear function [16–19]. The underlying reasons for non-linear effects are uncertain, though it may be that the risk of certain conditions does not increase significantly until a critical threshold of adversity has been exceeded [20].

The present study attempts to advance the literature by examining the effects of ACEs on reproductive health in a low-income sample of women. We hypothesized that our analyses would uncover a dose-response relationship between ACEs and three birth outcomes: (1) pregnancy loss, (2) preterm birth, and (3) low birthweight. In addition to testing linear associations, we explore whether the relationship between ACEs and birth outcomes is non-linear, the hypothesis being that effects are observable only once participants are exposed to a high number of ACEs.

## Methods

### Study and Sample Design

The present study is a secondary analysis of longitudinal data collected from low-income women with children in Wisconsin, United States. All participants received services within a statewide network of evidence-based home visiting programs that are supported by the federal Maternal Infant and Early Childhood Home Visiting Program [21]. Agencies in the network serve women who are pregnant or recently gave birth and who meet one or more risk factors (e.g., household poverty, substance use). Approximately 98% of the women served were at or below 200% of the federal poverty line or were eligible for federal means-tested benefits.

This investigation uses child and caregiver data that are collected by home visiting personnel and entered into a state-administered public health database. During routine prenatal and postpartum assessments, home visitors routinely gather information about client ACEs, pregnancy history, and birth outcomes. The study sample is composed of 1848 women who received home visiting services between July 2015 and January 2018. Women were included in the sample if they (a) were at least 16 years old at program enrollment, (b) completed an assessment of ACEs with home visiting staff, and (c) had valid prenatal and postpartum assessment records. Access to participant records was granted by the Wisconsin Department of Children and Families pursuant to a data sharing agreement and approval by a university institutional review board.

### Measures

#### Birth outcomes

We used archival program records to measure three dichotomous indicators of reproductive health: (1) any pregnancy loss, (2) any premature birth, and (c) any low birthweight infant. A measure of any pregnancy loss denotes whether a sample member ever had a miscarriage (i.e., pregnancy loss < 20 weeks gestation) or stillbirth (i.e., pregnancy loss > 20 weeks gestation). Premature birth indicates whether a participant ever gave birth prior to the 37th week of pregnancy. Low birthweight indicates whether a participant ever gave birth to an infant weighing less than 2500 g, or 5.5 pounds.

#### Adverse childhood experiences (ACEs)

Self-reported ACE histories were collected from participants by home visiting staff, typically within 90 days of program enrollment, using the Childhood Experiences Survey, a 19-item assessment that has demonstrated good internal consistency, test-retest reliability, and predictive validity [22]. Following conventions in the literature, a total ACE score was created by summing 10 dichotomous ACE indicators: physical abuse, sexual abuse, emotional abuse, physical neglect, emotional neglect, household substance abuse, household mental illness, household crime, domestic violence, and divorce/separation. We also created mutually exclusive groups based on the number of ACEs each participant reported: (1) no ACEs, (2) one or two ACEs, (3) three or four ACEs, and (4) five or more ACEs.

#### Covariates

All multivariate analyses included participant age, race/ethnicity, and educational attainment as covariates. Age was calculated at the date when home visiting staff gathered reproductive health data during a prenatal assessment. Race/ethnicity was coded into five categories, including Hispanics and four non-Hispanic groups: American Indian; African American, Caucasian, and Other race/ethnicity. Educational attainment was measured as dichotomy indicating if participants had any record of postsecondary education, meaning that they had received at least one college course credit or vocational training after high school.

### Statistical analyses

A descriptive analysis was performed to assess the mean, standard deviation, and frequency of study variables. Next, multivariate logistic regressions were conducted to test whether cumulative ACE scores were associated with a linear increase in the risk of pregnancy loss, preterm birth, and low birthweight while controlling for maternal age, race/ethnicity, and education. We then disaggregated the total ACE score into categorical predictors, as described in the measures section above. Multivariate analyses were repeated whereby independent groups of participants with 1–2 ACEs, 3–4 ACEs, or 5 or more ACEs were compared to a reference group with 0 ACEs. All analyses were conducted using IBM SPSS 25 statistical software.

## Results

Table 1 presents descriptive values for sample characteristics and key study variables. The mean age of participants was 25.5 (*SD* = 5.9). The racial/ethnic composition of the sample was 41.1% Caucasian, 24.0% African American, 23.2% Hispanic, 7.1% American Indian, and 4.9% Other race/ethnicity. Approximately 29.7% of participants had completed some postsecondary education. On average, participants endorsed 3.2 ACEs (*SD* = 2.6); 84.4% of subjects reported at least one ACE, 68.2% reported two or more ACEs (not shown), and 29.8% reported 5 or more ACEs. Results showed that 27.3% participants had at least one pregnancy loss, 14.8% of women had given birth prematurely, and 12.0% had given birth to a low birthweight infant.

**Table 1:** Sample Characteristics (*N* = 1848)

Study Measures	Mean (SD) or n (%)
Demographic Characteristics	
Age	25.5 (5.9)
Any postsecondary education	549 (29.7)
Race/Ethnicity	
American Indian	131 (7.1)
Caucasian	760 (41.1)
African American	444 (24.0)
Hispanic	428 (23.2)
Other race/ethnicity	91 (4.9)
Adverse Childhood Experiences	
Total ACE score	3.2 (2.6)
0 ACEs	288 (15.6%)
1 or 2 ACEs	557 (30.1%)
3 or 4 ACEs	453 (24.5%)
5 or more ACEs	550 (29.8%)
Outcomes	
Any pregnancy loss	505 (27.3%)
Any preterm birth (> 3 weeks early)	270 (14.8%)
Any low birthweight (< 5.5 pounds)	220 (12.0%)

*Abbreviation*: *ACE* Adverse childhood experience

Multivariate analyses presented in Table 2 indicated that, as hypothesized, the 10-item ACE index was associated with a significant increase in the odds of having experienced a pregnancy loss (OR = 1.12; 95% CI = 1.08–1.17). Higher ACE scores also were significantly associated with an increased likelihood of preterm birth (OR = 1.07; 95% CI = 1.01–1.12) and low birthweight (OR = 1.08; 95% CI = 1.03–1.15). Put another way, the results suggest that each additional ACE was associated with a 12% increase in the odds of pregnancy loss, a 7% increase in the odds of preterm birth, and an 8% increase in the odds of low birthweight.

**Table 2:** Reproductive health outcomes regressed on adverse childhood experiences

	Any pregnancy loss (*n* = 1848)		Any preterm birth (*n* = 1823)		Any low birthweight (*n* = 1837)	
	Model 1 OR (95% CI)	Model 2 OR (95% CI)	Model 1 OR (95% CI)	Model 2 OR (95% CI)	Model 1 OR (95% CI)	Model 2 OR (95% CI)
Age	1.07** (1.05–1.09)	1.07** (1.05–1.08)	1.08** (1.05–1.10)	1.08** (1.05–1.10)	1.06** (1.03–1.08)	1.06** (1.03–1.08)
Race/Ethnicity^1^						
American Indian	0.65 (0.41–1.02)	0.66 (0.42–1.04)	0.63 (0.35–1.12)	0.64 (0.36–1.14)	0.53 (0.28–1.00)	0.55 (0.29–1.03)
Caucasian	0.93 (0.71–1.22)	0.94 (0.72–1.23)	0.83 (0.60–1.16)	0.85 (0.61–1.19)	0.60* (0.42–0.85)	0.61* (0.43–0.87)
Hispanic	0.70* (0.51–0.96)	0.69* (0.51–0.95)	0.51* (0.34–0.77)	0.51* (0.34–0.77)	0.49* (0.32–0.74)	0.48 (0.32–0.74)
Other race/ethnicity	0.65 (0.37–1.12)	0.63 (0.36–1.09)	0.67 (0.34–1.32)	0.67 (0.34–1.31)	0.75 (0.38–1.47)	0.73 (0.37–1.42)
Any postsecondary education	1.05 (0.83–1.32)	1.05 (0.83–1.32)	0.73* (0.54–0.99)	0.73* (0.54–0.98)	0.84 (0.61–1.16)	0.84 (0.61–1.16)
Total ACE score	1.12** (1.08–1.17)		1.07** (1.01–1.12)		1.08** (1.03–1.15)	
1 or 2 ACEs		0.93 (0.66–1.31)		1.22 (0.79–1.89)		0.98 (0.62–1.56)
3 or 4 ACEs		1.27 (0.89–1.80)		1.29 (0.82–2.02)		1.22 (0.76–1.96)
5 or more ACEs		1.80** (1.28–2.52)		1.46 (0.95–2.26)		1.39 (0.88–2.19)

*Abbreviations*: *OR* odds ratio, *CI* Confidence interval. *p < .05; **p < .01

When we modeled ACEs as categorical indicators, results showed that there were no significant differences in birth outcomes between participants with 0 ACEs and participants with 1–2 ACEs or 3–4 ACEs. Having 5 or more ACEs was associated with an increase in the odds of pregnancy loss (OR = 1.80; 95% CI = 1.28–2.52) while associations with preterm birth (OR = 1.46; 95% CI = 0.95–2.26) and low birthweight (OR = 1.39; CI = 0.88–2.19) did not reach statistical significance.

**Fig. 1:**
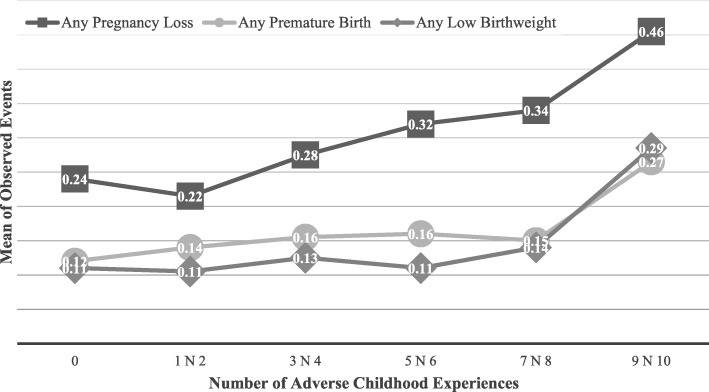
Mean of observed negative birth outcomes by the number of adverse childhood experiences

Figure 1 shows the unadjusted (i.e., observed) means for all three study outcomes based on the number of ACEs endorsed. The relationship between cumulative ACE scores and pregnancy loss appeared to be approximately linear, while associations between ACEs and both preterm birth and low birthweight appeared to deviate from linearity. To formally test for non-linear effects, we ran supplemental analyses that added a quadratic term to the multivariate logistic regressions. Results showed that the quadratic term in the low birthweight model was significant (OR = 1.02, 95% CI = 1.00–1.04), suggesting the relationship was non-linear. The quadratic term was not significant in either the pregnancy loss (OR = 1.01, 95% CI = 0.99–1.02) or preterm birth models (OR = 1.01, 95% CI = 0.99–1.03).

## Discussion

Results from this study indicated that a large majority (84.4%) of low-income women receiving home visiting services had at least one ACE, and over two-thirds (68.2%) reported multiple ACEs. We also confirmed that elevated levels of childhood adversity undermine reproductive health, as higher ACE scores were associated with a greater likelihood of pregnancy loss, preterm birth, and low birthweight. Multivariate analyses showed that each additional ACE was associated with a 12% increase in the odds of pregnancy loss, a 7% increase in the odds of preterm birth, and an 8% increase in the odds of low birthweight. Taken at face value, the findings point to a dose-response relationship between the number of adverse childhood experiences and the risk of adverse birth outcomes.

When ACEs were modeled as categorical groupings rather than as a cumulative index, however, a more nuanced pattern of association emerged. The observed relationship between ACE scores and pregnancy loss appeared to follow a largely continuous function, whereas associations between ACE scores and both preterm birth and low birthweight clustered at the most extreme levels of adversity. Multivariate analyses confirmed that the relationship between a total ACE score and low birthweight fit a quadratic model, denoting a significant deviation from linearity. The results suggest that ACEs are associated generally with poor birth outcomes, but that certain outcomes may only manifest once an individual has been exposed to profound adversity. Supporting this interpretation, a long line of research on cumulative risk has sometimes uncovered similar threshold effects [20, 23].

A corollary to the above explanation is that outcomes may differ in sensitivity to less extreme gradations of adversity. One potential reason is that the relative proportion of variance explained by genetic and environmental causes differ across outcomes [24]. A significant amount of variability in preterm birth, for example, is attributable to genetic factors [25]. Theoretically, as the proportion of environmental influence on a given outcome decreases, the magnitude of effect associated with ACEs must increase to alter the outcome. This could partly explain why the effects of ACEs on preterm birth and low birthweight appeared to be concentrated at the highest levels of adversity.

The impact of ACEs also may vary by the developmental timing of the outcome. Other studies have documented robust associations between ACE scores and poor outcomes in later life, whereas we uncovered statistically significant, yet comparatively modest associations between a cumulative ACE index and reproductive health outcomes in early adulthood. It is possible that the effects of ACEs on physical health may increase over time due to the wear and tear of stress processes that are catalyzed by early adversity [26, 27]. The effects of ACEs may accrue over time through psychosocial mechanisms as well [28].

The results also should be interpreted considering certain methodological features of the study, including the low-income sampling frame. Participants who were exposed to few ACEs still may have experienced poor birth outcomes due to their experiences of poverty and other associated risks [29, 30]. As a result, the estimated magnitude of ACEs on birth outcomes may be smaller than those that would likely emerge from a more generalizable or advantaged sample [31]. In addition, cumulative ACE scores are imprecise instruments that do not account for the discrete effects of specific ACEs or the combinative effects of certain ACE constellations. Additive ACE scores ignore various aspects of adversity such as its type, timing, or severity, which may contribute to differential health outcomes. In addition, future ACE research will be advanced by measuring adversity prospectively and by measuring more precise birth outcomes, including specific thresholds of preterm birth (e.g., moderate-to-late; very; extremely) and low birthweight (e.g., very; extremely). Moreover, ACEs and birth outcomes were measured using self-report data, which may introduce measurement error due to underreporting or misreporting. Finally, our statistical models did not include variables that could otherwise account for the observed effects of ACEs, including genetic, epigenetic, biological, psychological, and behavioral factors.

## Conclusion

The current study adds to emerging evidence that ACEs are deleterious to reproductive health. Results showed that exposure to a greater number of ACEs increased the risk of pregnancy loss, preterm birth, and low birthweight. The relationship between ACEs and pregnancy loss largely followed a dose-response pattern, while the associations between ACEs and both preterm birth and low birthweight appeared to be at least partly non-linear. The latter findings signify potential threshold effects, meaning that some poor birth outcomes may emerge only when an individual is exposed to a critical level of adversity. The findings also justify further inquiry into non-additive and interactive effects of ACEs as well as the extent to which the timing, frequency, severity, and duration of adverse experiences yield differential outcomes.

Our work also adds to a growing interest in applying a life course perspective to the study of maternal and child health [32, 33]. A large body of research has shown that birth outcomes can be affected by proximal stressors such as domestic violence [33], yet few studies have examined whether adverse and traumatic events in childhood are associated with similar consequences. In addition to investigating main-effect associations, we encourage other researchers to explore the causal mechanisms through which ACEs lead to poor birth outcomes. For example, ACEs may affect reproductive health through any number of biological changes that are embedded during childhood, including compromised neuroendocrine and immune functions [33, 34]. Birth outcomes also may be influenced indirectly by ACEs through psychosocial pathways, including elevated levels of stress and anxiety [35–37], tobacco and substance use [38–40], and exposure to adverse adult experiences [28]. Insights into the processes through which adverse experiences lead to adverse birth outcomes may help to inform interventions such as home visiting programs that have the potential to mitigate the effects of ACEs and promote reproductive health.

## Data Availability

The datasets used and/or analyzed during the current study are available from the corresponding author on reasonable request.

## Abbreviations

ACE: Adverse childhood experience
CI: Confidence interval
OR: Odds ratio
